# Supplementary material for: Rhythmic oscillations in the midbrain dopaminergic nuclei in mice
Source: Front Cell Neurosci. 2023 Jun 23;17:1131313. doi: 10.3389/fncel.2023.1131313 (PMC10326437; doi:10.3389/fncel.2023.1131313)
Supplement: Supplementary file 1 [file Image_1.PDF]

A

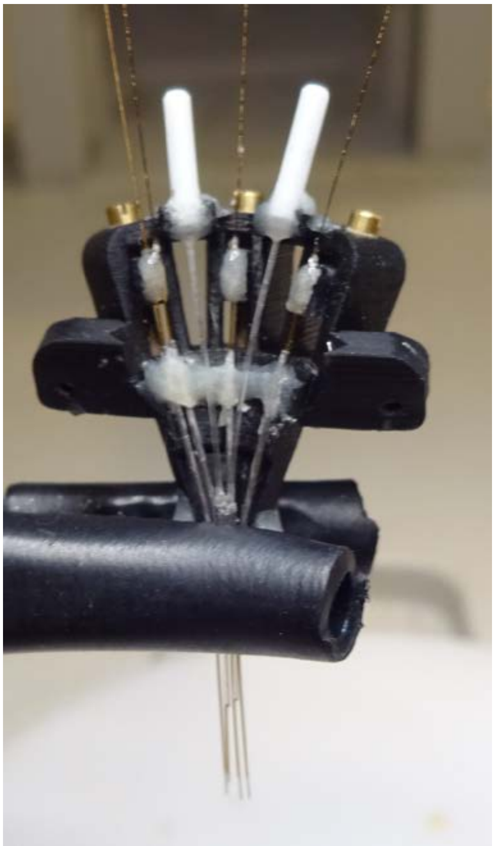

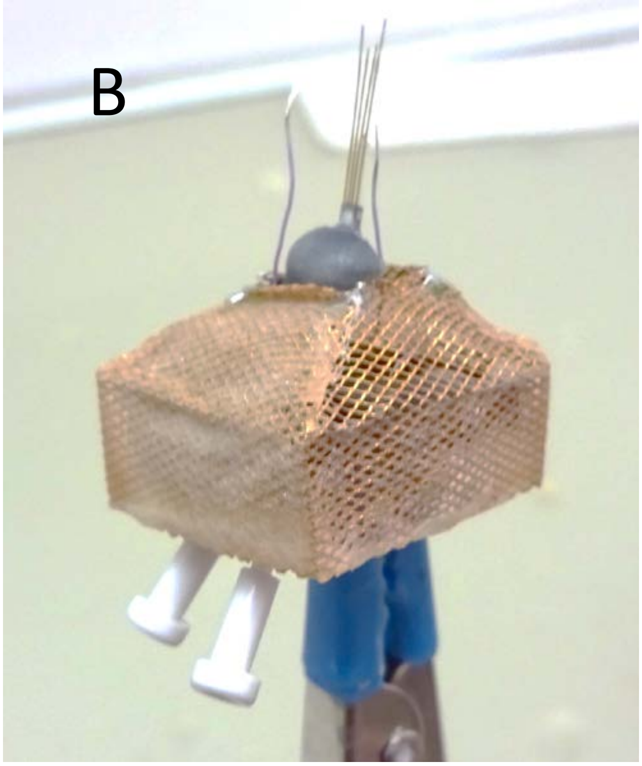

B

C

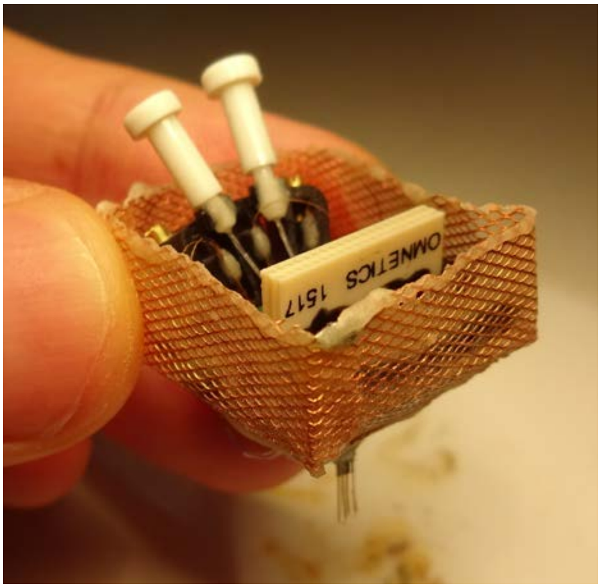

Supplementary Figure 1.
The optrode. A) Cutaway view. White cylinders are the ferrules with optic fibers descending. Brass screws drive the octrodes ventrally. B) The assembly with Faraday cage, and ground wires protruding above. C) The assembly with white connector interface and octrode wires cut to desired length, ready for implantation.
