# Supplementary material for: Rhythmic oscillations in the midbrain dopaminergic nuclei in mice
Source: Front Cell Neurosci. 2023 Jun 23;17:1131313. doi: 10.3389/fncel.2023.1131313 (PMC10326437; doi:10.3389/fncel.2023.1131313)
Supplement: Supplementary file 2 [file Image_2.PDF]

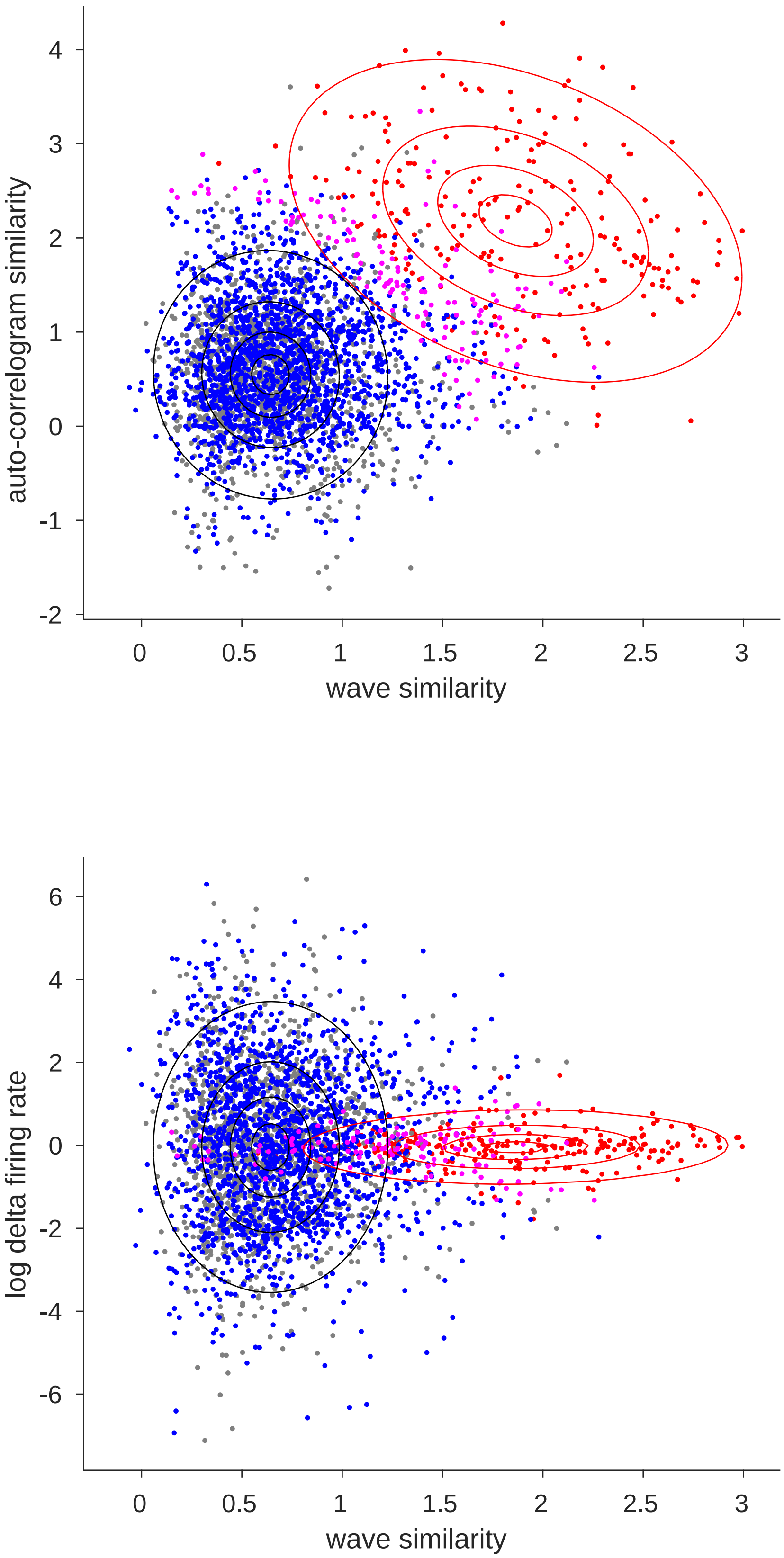

Supplementary Figure 2. Classification of different- and same-neuron pairs recorded in consecutive days. Each dot represents similarity scores of electrophysiological features of a pair of neurons recorded in consecutive days. Dark gray dots are pairs recorded from different octrodes; blue and red dots are pairs recorded from the same octrodes estimated as different- and same-neuron pairs, respectively; pink dots are uncategorized pairs. The estimated Gaussians of different- (blue) and same-neuron (red) pairs are shown as contour plots. The contours correspond to 25%, 50%, 75%, and 95% of each distribution.
