# Supplementary material for: Rhythmic oscillations in the midbrain dopaminergic nuclei in mice
Source: Front Cell Neurosci. 2023 Jun 23;17:1131313. doi: 10.3389/fncel.2023.1131313 (PMC10326437; doi:10.3389/fncel.2023.1131313)
Supplement: Supplementary file 3 [file Image_3.pdf]

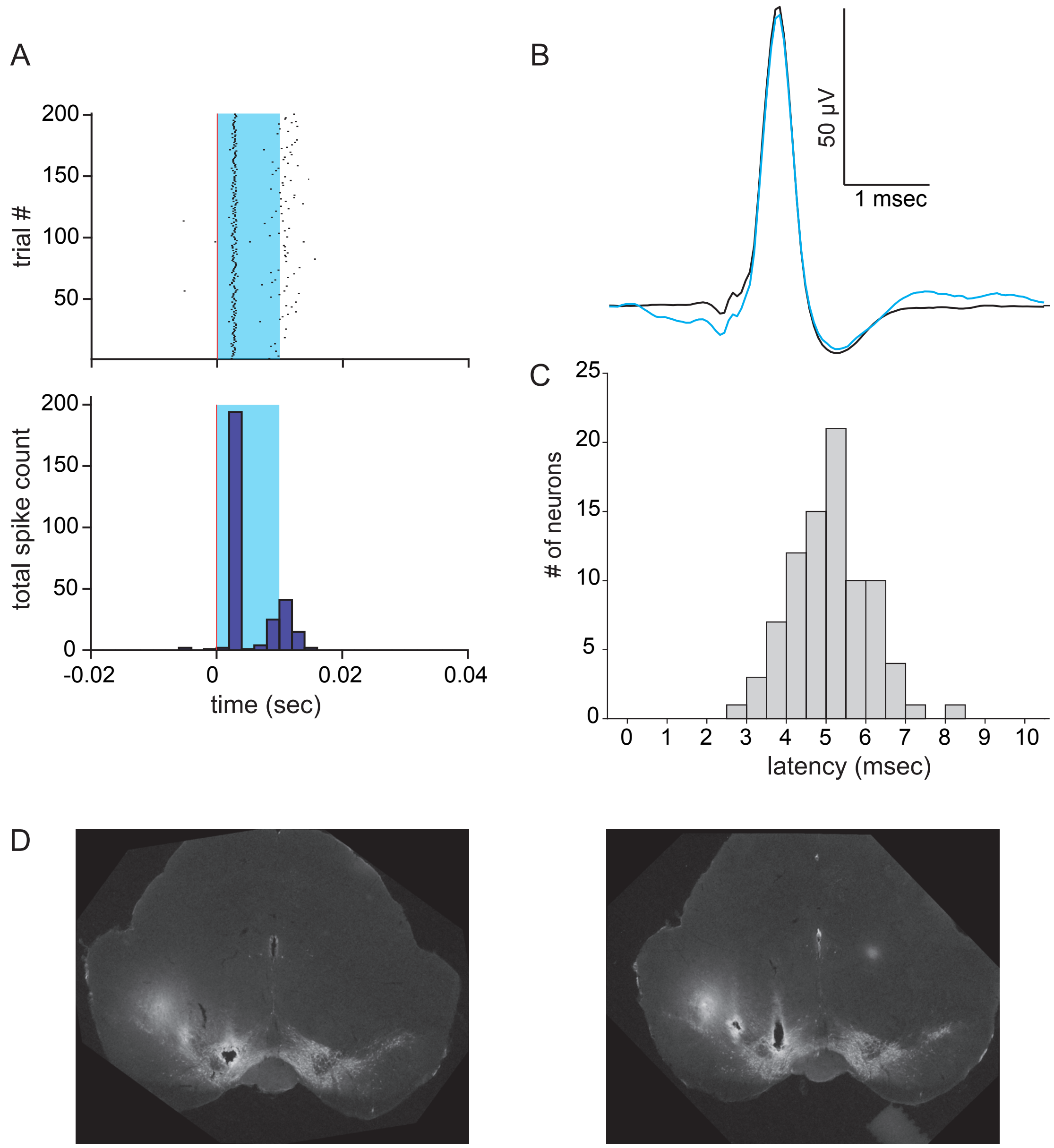

Supplementary Figure 3. Recordings in optogenetically identified dopaminergic nuclei in the transgenic mice. A) Example of a dopaminergic neuron response to light stimuli (highlighted in blue shaded period). Spikes appear above, and summary histograms below. B) Waveform of the neuron in A. C) Mean latencies of first spikes for all responsive neurons recorded. D) Example of tyrosine hydroxylase histochemistry of coronal brain sections with electrode tracks and electrolytic marking lesions (holes in tissue on left side). The lateral-most electrode did not reach the SNc in this mouse.
