# Supplementary material for: Rhythmic oscillations in the midbrain dopaminergic nuclei in mice
Source: Front Cell Neurosci. 2023 Jun 23;17:1131313. doi: 10.3389/fncel.2023.1131313 (PMC10326437; doi:10.3389/fncel.2023.1131313)
Supplement: Supplementary file 4 [file Image_4.pdf]

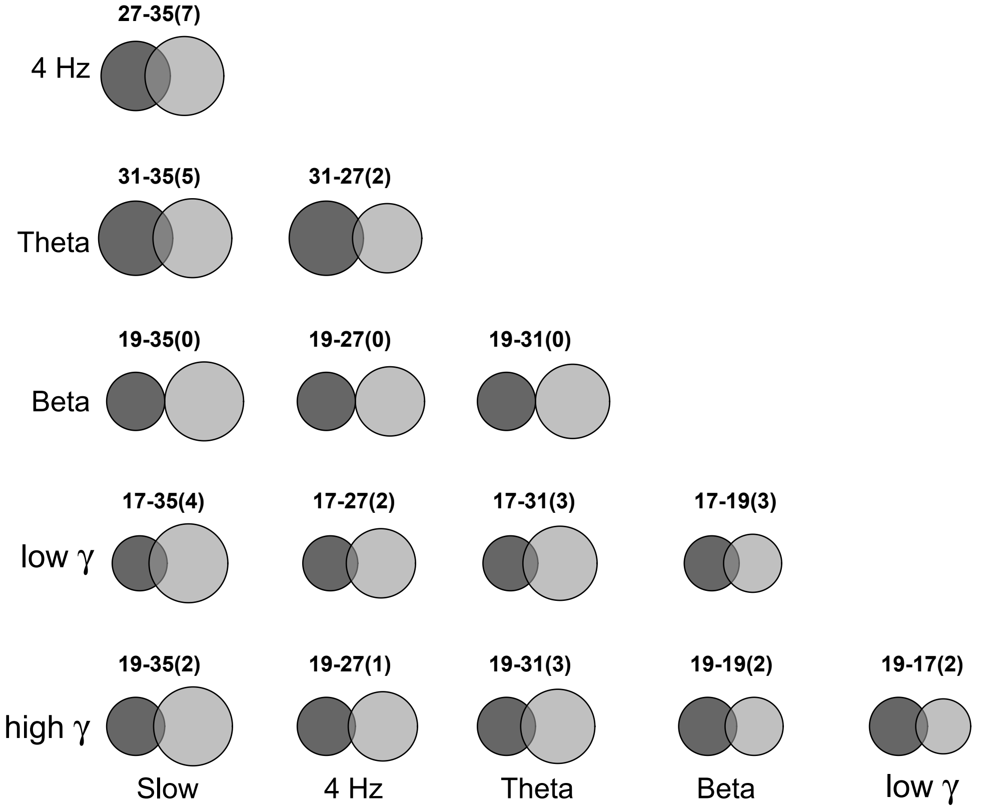

Supplementary Figure 4. Low incidence of significant phase-locking of the same neuron to two different frequency bands. Phase-locking was determined by PPC. Venn diagrams indicate the numbers of neurons phase-locked to the bands listed to the left by the area of the black circles, and phase-locked to the bands listed below by the area of the light gray circles. Overlap (dark gray) indicates the low numbers of neurons phase-locked to both bands. Respective numbers are shown above, with overlap in parentheses.
