# Supplementary material for: Rhythmic oscillations in the midbrain dopaminergic nuclei in mice
Source: Front Cell Neurosci. 2023 Jun 23;17:1131313. doi: 10.3389/fncel.2023.1131313 (PMC10326437; doi:10.3389/fncel.2023.1131313)
Supplement: Supplementary file 5 [file Image_5.pdf]

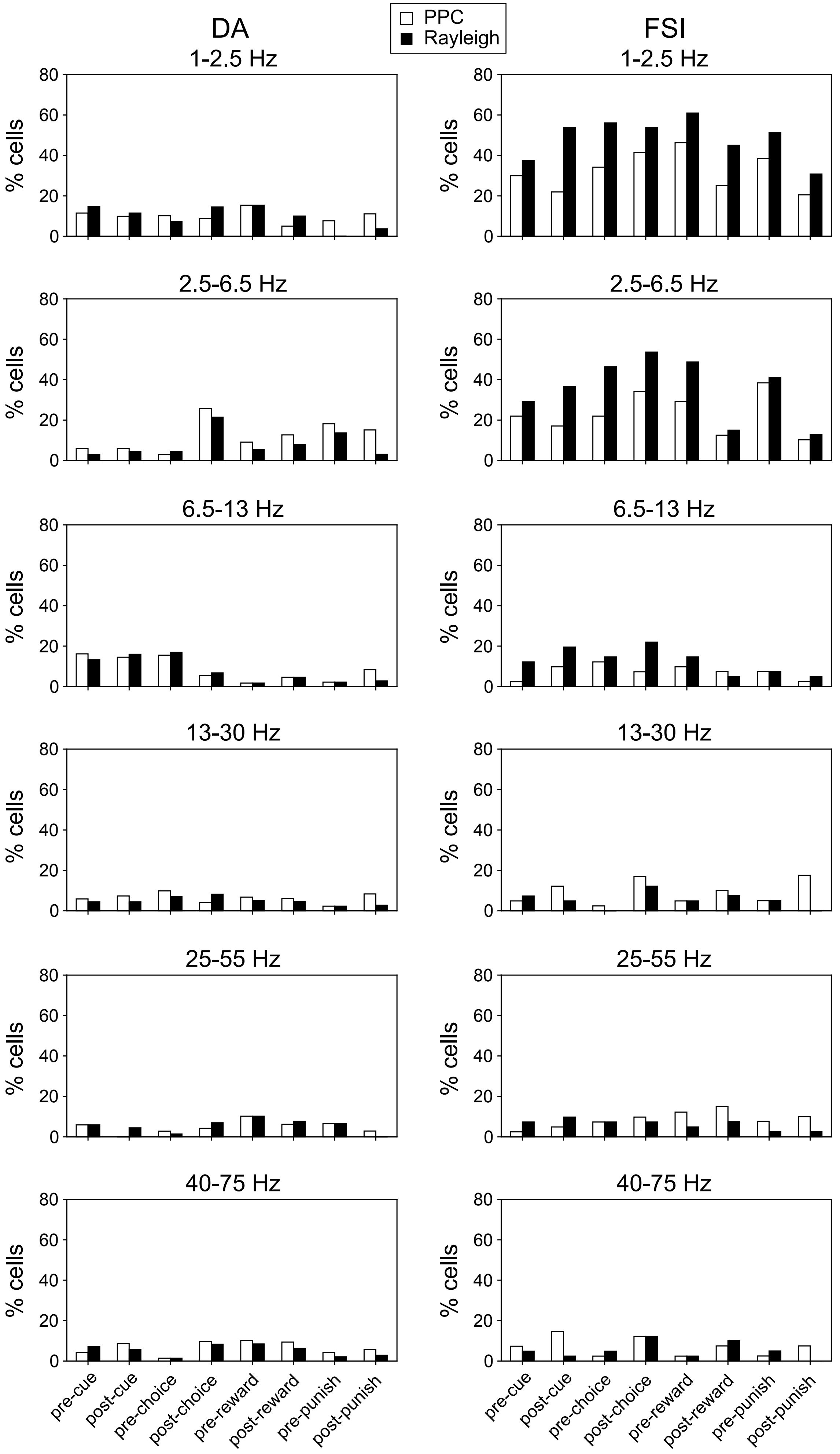

Supplementary Figure 5. Similar profiles in Rayleigh test and PPC for significant phase locking in neurons recorded in the midbrain dopaminergic nuclei during the behavioral task.
